# IGFBP-1 Expression Promotes Tamoxifen Resistance in Breast Cancer Cells via Erk Pathway Activation

**DOI:** 10.3389/fendo.2020.00233

**Published:** 2020-05-06

**Authors:** Yan Zheng, Janel Y. Sowers, Kevin D. Houston

**Affiliations:** Department of Chemistry and Biochemistry, New Mexico State University, Las Cruces, NM, United States

**Keywords:** tamoxifen, breast cancer, drug resistance, IGFBP-1, EGFR

## Abstract

Insulin-like growth factor (IGF) system plays a significant role in many cellular processes, including proliferation, and survival. In estrogen receptor positive breast cancer, the level of circulating IGF-1 is positively associated with the incidence and at least 50% of cases have elevated IGF-1R signaling. Tamoxifen, a selective estrogen receptor modulator and antagonist for estrogen receptor alpha (ERα) in breast tissue, is a commonly prescribed adjuvant treatment for patients presenting with ERα-positive breast cancer. Unfortunately, tamoxifen resistance is a frequent occurrence in patients receiving treatment and the molecular mechanisms that underlie tamoxifen resistance not adequately defined. It has recently been reported that the inhibition of IGF-1R activation and the proliferation of breast cancer cells upon tamoxifen treatment is mediated by the accumulation of extracellular insulin-like growth factor binding protein 1 (IGFBP-1). Elevated IGFBP-1 expression was observed in tamoxifen-resistant (Tam^R^) MCF-7 and T-47D cells lines suggesting that the tamoxifen-resistant state is associated with IGFBP-1 accumulation. MCF-7 and T-47D breast cancer cells stably transfected with and IGFBP-1 expression vector were generated (MCF7-BP1 and T47D-BP1) to determine the impact of breast cancer cell culture in the presence of increased IGFBP-1 expression. In these cells, the expression of IGF-1R was significantly reduced compared to controls and was similar to our observations in tamoxifen-resistant MCF-7 and T-47D cells. Also similar to Tam^R^ breast cancer cells, MCF7-BP1 and T47D-BP1 were resistant to tamoxifen treatment, had elevated epidermal growth factor receptor (EGFR) expression, increased phospho-EGFR (pEGFR), and phospho-Erk (pErk). Furthermore, tamoxifen sensitivity was restored in the MCF7-BP1 and T47D-BP1 upon inhibition of Erk phosphorylation. Lastly, the transient knockdown of IGFBP-1 in MCF7-BP1 and T47D-BP1 inhibited pErk accumulation and increased tamoxifen sensitivity. Taken together, these data support the conclusion that IGFBP-1 is a key component of the development of tamoxifen resistance in breast cancer cells.

## Introduction

Insulin-like growth factor (IGF) signaling is a complex system that affects almost every organ in the human body via regulation of multiple cellular processes, such as proliferation, survival, mitogenesis, migration, senescence, angiogenesis, and autophagy (1, 2). The IGF system consists of two natural ligands, insulin-like growth factor-1 (IGF-1) and IGF-2; two transmembrane receptors, insulin-like growth factor 1 receptor (IGF-1R) and IGF-2R; and six high affinity IGF binding proteins (IGFBPs) 1-6 (3). The binding of IGF-1 or IGF-2 to IGF-1R results in the activation of tyrosine kinase activity of the receptor (4), which in turn activates phosphatidylinositol 3-kinase (PI3K)-AKT pathway and mitogen-activated protein kinases (MAPK) pathway (5). IGF-2R, on the other hand, acts as a tumor suppressor and directs the degradation of IGF-2 specifically (6). The bioavailability and half-life of IGF-1 and IGF-2 are tightly regulated by IGFBP1-6 (7), each of which has different binding affinities and distinct functions depending on the tissue (8). In addition to the complexity of the IGF system, there is an increasing body of evidence showing the interactions between IGF pathway and other hormone signaling pathways suck as estrogen receptor (ER) pathway (9) and epidermal growth factor receptor (EGFR).

The IGF system plays an important role in breast cancer as exemplified both *in vitro* and *in vivo* (10). At least 50% of breast tumors present with activated IGF-1R (11) and the level of circulating IGF-1 positively correlates with the incidence of estrogen receptor positive (ER positive) breast tumors (3). The tumor volume was significantly higher in the xenografts containing ER positive MCF-7 cells with IGF-1 overexpression compared to the control in the mouse model (12); IGF-1 potentiated the invasive ability of MCF-7 cells (13). IGFBP-1, inhibitor of IGF-1 signaling, decreases activation of IGF-1R and inhibits proliferation and survival in MCF-7 cells (14).

Tamoxifen, a selective estrogen receptor modulator and antagonist for estrogen receptor alpha (ERα) is a commonly prescribed adjuvant treatment for patients presenting with ERα-positive breast cancer. IGFBP-1 has also been shown to mediate the decrease in cell viability observed in tamoxifen-treated MCF-7 cells (15). In spite of the clinical benefit of tamoxifen treatment, about 40% of the patients develop resistance to tamoxifen over the course of treatment (16). It has been found that the loss of IGF-1R expression is one of most significant characteristics of acquired tamoxifen resistance (17). As a result, it was hypothesized that the accumulation of IGFBP-1 upon long-term tamoxifen treatment would result in the loss of IGF-1R expression, and eventually lead to the development of tamoxifen resistance.

In this study, initially we discovered that both tamoxifen resistant MCF-7 and T-47D cells expressed higher level of IGFBP-1 compared to parental cells. Then we found that both IGFBP-1 overexpressing MCF-7 and T-47D (MCF7-BP1 and T47D-BP1) cells shared some similarities with the corresponding TamR cells, such as the reduction of IGF-1R expression and increased Erk phosphorylation. Furthermore, we shown that both MCF7-BP1 and T47D-BP1 were tamoxifen-nonresponsive. Moreover, we found the transient knockdown of IGFBP-1 expression in these stable cells resulted in the reduced level of pErk and re-sensitized the cells to tamoxifen. Finally, we demonstrated the transient knockdown of IGFBP-1 restored the tamoxifen sensitivity in MCF7-TamR and T47D-TamR cells. Taken together, our data revealed a new mechanism of tamoxifen action that contributed to the development of tamoxifen resistance.

## Materials and Methods

### Cell Culture

MCF-7 and T-47D breast cancer cells were purchased from ATCC (ATCC, Manassas, VA). All cells lines were maintained in maintenance DMEM supplemented with 10% fetal bovine serum, 1 mM sodium pyruvate and 2 mM L-glutamine (Life Technologies, Carlsbad, CA). All cell lines for experiments were lower than passages 35 and both nucleotide and protein purifications were performed on cell lines at similar confluency.

### Establishment of Stably Transfected Cells

Human IGFBP-1 expression vector (NM_000596) and the vector devoid of *IGFBP-1* ORF were purchased from OriGene (Rockville, MD). Plasmid transfection was performed using Lipofectamine 3000 reagent in serum-free Opti-MEM (Life Technologies, Carlsbad, CA) according to the manufacture's protocol. After 96 h of transfection, cells were washed with 1X PBS, and allowed to recover in maintenance media for 24 h then washed with 1X PBS followed by the addition of fresh maintenance media containing 800 or 400 μg/mL Geneticin (Life Technologies, Carlsbad, CA) for MCF-7 and T-47D cells, respectively. Untransfected cells were treated with Geneticin every 5 days until all cells were killed to demonstrate efficacy of Geneticin. All stably transfected cells were validated after selection by immunoblot and qRT-PCR. The stably transfected cell lines with IGFBP-1 containing plasmid were named MCF7-BP1 or T47D-BP1 and cells containing the vector devoid of *IGFBP-1* ORF were named MCF7-EV and T47D-EV.

### Establishment of Tamoxifen Resistance (TamR) Cells

The method of establishing tamoxifen resistance cells was previously described (18). Briefly, cells were exposed 1 μM 4-hydroxytamoxifen (4-OHT) (Fluka, St. Louis, MO) in maintenance media. After 72 h of exposure, spent media was removed and new maintenance media containing 1 μM 4-OHT was added. After 21 days of 4-OHT exposure, cells that remained were allowed to recover and grow in fresh maintenance media. Cells were then split and maintained in maintenance media containing 1 μM 4-OHT. The cell lines generated by this method were named MCF7-TamR and T47D-TamR.

### shRNA Knockdown

Human IGFBP-1 shRNA plasmid kit (Locus ID 3484) was purchased from OriGene (Rockville, MD). Plasmid transfection was performed using Lipofectamine 3000 reagent in serum-free Opti-MEM (Life Technologies, Carlsbad, CA) according to the manufacture's protocol. After 96 h of shRNA knockdown, cells were harvested and the expression of IGFBP-1 was measured by immunoblot.

### Cell Treatment

4-hydroxytamoxifen (4-OHT) (Sigma-Aldrich, St. Louis, MO) treatment was previously described (15). Briefly, 48 h prior to the treatment, cells were washed with 1X PBS and maintenance media was replaced with phenol red-free DMEM supplemented with 1% charcoal-stripped FBS (CS media) (Life Technologies, Carlsbad, CA). Cells were then washed with 1X PBS and treated with indicated concentrations of 4OHT in serum-free DMEM for 5 days. Ethanol was used to dissolve 4OHT. PD98059 (Life Technologies, Carlsbad, CA) was used to block the activation of MAP kinase (MEK). Forty eight hours prior to the treatment, cells were washed with 1X PBS and maintenance media was replaced with phenol red-free DMEM supplemented with 1% charcoal-stripped FBS (CS media) (Life Technologies, Carlsbad, CA). Cells were then washed with 1X PBS and treated with indicated concentrations of PD98059 in serum-free DMEM for 5 days. Ethanol was used to dissolve PD98059. For the EGF treatment, recombinant human EGF (Life Technologies, Carlsbad, CA) was used. Forty eight hours prior to the treatment, cells were washed with 1X PBS and maintenance media was replaced with phenol red-free DMEM supplemented with 1% charcoal-stripped FBS. After 24 h, cells were washed with 1X PBS and starved with serum and phenol red-free DMEM. After 24 h, cells were washed with 1X PBS and treated with serum and phenol red-free DMEM with the addition of indicated amount of EGF. Deionized water was used to dissolve the lyophilized EGF. Cells were harvested in 5 days.

### Total RNA Extraction and Quantitative Real-Time PCR Analysis

Total RNA was extracted using the PureLink RNA Mini Kit (Life Technologies, Carlsbad CA) followed by on-column DNA digestion using Purelink DNase Set (Life Technologies, Carlsbad CA). cDNA was synthesized from 1 μg total RNA using the High Capacity RNA-to-cDNA Kit (Life Technologies, Carlsbad CA) and used as template in subsequent quantitative real-time PCR (RT-qPCR) reactions. qRT-PCR was performed using SYBR Green Master Mix (Life Technologies, Carlsbad CA) and the 7300 Real-Time PCR system (Bio-Rad, Hercules, CA). Primer pairs used for qRT-PCR: human IGFBP-1 forward 5′-CTA-TGA-TGG-CTC-GAA-GGC-TC-3′; reverse 5′-TTC-TTG-TTG-CAG-TTT-GGC-AG-3′ (19). Human IGF-1R forward 5'-GCA-CCA-TCT-TCA-AGG-GCA-ATT-TG-3′; reverse 5′-AGG-AAG-GAC-AAG-GAG-GAC-CAA-GG-3′. Human RPL30 gene was used as the internal control to normalize for mRNA in qRT-PCR reactions. Human RPL30 forward 5′-ACA-GCA-TGC-GGA-AAA-TAC-TAC-3′; reverse 5′-AAA-GGA-AAA-TTT-TGC-AGG-TTT-3′ (20).

### Immunoblot Analysis

To prepare samples for immunoblot analysis, cells were harvested with RIPA lysis buffer containing protease and phosphatase inhibitor cocktails (Prod# 89901, 1862209, and 186249, Thermo Scientific, Rockford, IL). After lysis, cells were centrifuged at 12,000 × g for 15 min at 4°C, supernatant was collected protein concentrations was determined by BCA assay (Thermo Scientific, Rockford, IL). 30–75 μg total protein was resolved using Bolt 4–12% Bis-Tris Plus gels and transferred to PVDF membrane (Life Technologies, Carlsbad, CA). PVDF membranes were blocked in 1X Tris-buffered saline-0.1% Tween 20 (TBST) containing 5% fat-free milk at room temperature for 1 h with slow agitation. Membranes were then washed with 1X TBST three times and primary antibody was added and allowed to incubate overnight at 4°C. The following primary antibodies including dilution factor in 5% milk TBST were used in the current study: IGFBP-1 (#31025, Cell Signaling Technology, Danvers, MA); IGF-1R (#3027, Cell Signaling Technology Danvers, MA); P-IGF-1R (Tyr 1131) (#3021, Cell Signaling Technology, Danvers, MA); p44/42 MAPK (Erk1/2) (#9102, Cell Signaling Technology, Danvers, MA); P-p44/42 MAPK (T202/204) (#4377, Cell Signaling Technology, Danvers, MA); EGFR (#4267, Cell Signaling Technology, Danvers, MA); P-EGFR (Tyr 1068) (#3777, Cell Signaling Technology, Danvers, MA); Integrin β1 (sc-374429, Santa Cruz Biotechnology, Dallas, TX); β-actin (sc-47778, Santa Cruz Biotechnology, Dallas, TX). The dilution ratio for primary antibodies from Cell Signaling Technology was 1:1,000; The dilution ratio for primary antibodies from Santa Cruz Biotechnology was 1:2,000. After primary antibody incubation, membranes were washed three times with 1X TBST then incubated with anti-rabbit IgG conjugated to horseradish peroxidase (#7074, Cell Signaling Technology, Danvers, MA) or anti-mouse IgG conjugated to horseradish peroxidase (sc-81178, Santa Cruz Biotechnology, Dallas, TX) with dilution ratio of 1:5,000 at room temperature for 1 h. After washing membranes with 1X TBST three times, chemiluminescence reagent (34076, Thermo Scientific, Rockford, IL) was added and detected using Gel Doc™ XR ChemiDoc™ imaging system (BioRad, Hercules, CA) followed by quantification using ImageJ (NIH). Restore plus western blot buffer (46430, Thermo Scientific, Rockford, IL) was used to strip membranes of antibodies prior to probing for loading control where needed.

### Extracellular IGFBP-1 Measurement

The method was previously described (15). Briefly, media was collected and concentrated with centrifugal filter units (UFC800396, MillporeSigma, Burlington, MA) at 4°C with the speed of 4,000 rpm for 1 h. Once centrifugated, media was collected with an addition of protease inhibitor cocktail (Prod #1862209, Thermo Scientific, Rockford, IL). Total protein concentration of concentrated media was measured by BCA assay, and the level of extracellular IGFBP-1 was determined by immunoblot analysis as previously described. For the external loading control, same amount of total protein (30 μg) of concentrated media samples were resolved by Bolt 4–12% Bis-Tris Plus gels. The gels were then washed with deionized water for 5 min and stained with Coomassie blue for 1 h. Thereafter, gels were destained with deionized water overnight. Gels were then imaged with FOTODYNE gel imager (FOTODYN INCORPORATED, Hartland, WI). The same intensity of protein band indicated the equivalent loading of samples.

### Cell Viability Assay

After 5 days of treatments, cells were trypsinized and harvested with 1X PBS. The cell numbers were determined by counting via hemocytometer and compared to the vehicle-treated samples, which were normalized to 100%.

### Statistical Analysis

All statistical analysis was performed by one-way ANOVA, Tukey's *post-hoc* test using Prism 6 (GraphPad, San Diego, CA). Differences were considered significant if *p* ≤ 0.05 and the error bars are ± SEM.

## Results

### MCF7-TamR and T47D-TamR Expressed More IGFBP-1 and the Establishment of MCF7-BP1 and T47D-BP1 Stable Cell Lines

**Figure 1:**
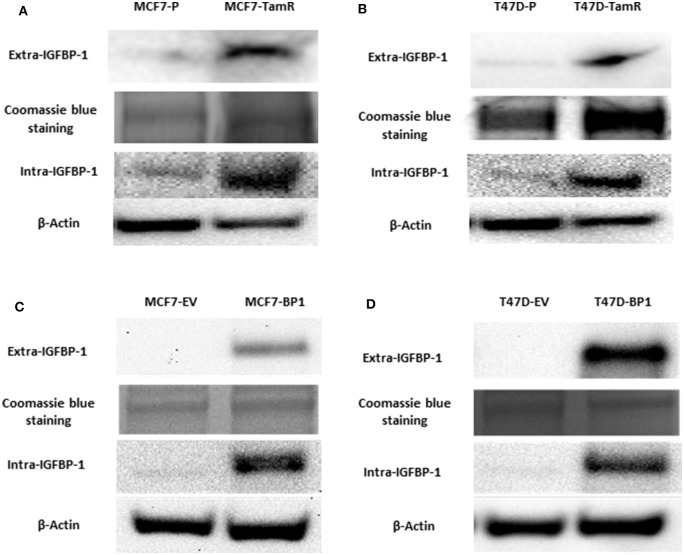
MCF7-TamR and T47D-TamR expressed more IGFBP-1 and the establishment of MCF7-BP1 and T47D-BP1 stable cell lines. Immunoblot analysis of IGFBP-1 expression in MCF-7 and T-47D stable cells. **(A)** Measurement of IGFBP-1 expression in MCF7-P and MCF7-TamR (left), and in T47D-P and T47D-TamR (right); **(B)** measurement of IGFBP-1 expression in MCF7-EV and MCF7-BP1 (left), and in T47D-EV and T47D-BP1 (right). The Coomassie blue staining indicated the even loading of the proteins from the concentrated media. Results are the representatives of 3 independent experiments. Extra-IGFBP-1: extracellular IGFBP-1; intra-IGFBP-1: intracellular IGFBP-1.

Previously, insulin-like growth factor binding protein-1 (IGFBP-1) induction in 4-hydroxytamoxifen (4-OHT)-treated breast cancer cells was shown to mediate the efficacy of 4-OHT (15). To determine if IGFBP-1 is critical for the development of tamoxifen resistance in breast cancer cells, the level of IGFBP-1 in MCF-7 parental cells (MCF7-P) and MCF-7 tamoxifen resistant cell (MCF7-TamR), as well as in T-47D parental (T47D-P) and T47D-TamR was determined. Both MCF7-TamR and T47D-TamR expressed higher levels of IGFBP-1 compared to parental cells that did not have detectable levels of IGFBP-1 (Figure 1A). These data suggested that IGFBP-1 may promote the development of tamoxifen resistance. To determine if IGFBP-1 exposure is sufficient for the development of tamoxifen resistance in breast cancer cells, MCF-7 and T-47D cells with stable overexpression of IGFBP-1 were generated. After selection, both extracellular and intracellular levels of IGFBP-1 were determined by immunoblot. For both MCF-7 and T-47D cells, the level of intracellular and extracellular IGFBP-1 in empty vector (EV) controls was low and similar to the parental cell lines. For MCF-7 and T-47D cells stably expressing the IGFBP-1 expression vector (designated BP1 for each cell line), high levels of both intracellular and extracellular IGFBP-1 were observed compared to the EV controls (Figure 1B). Additionally, MCF7-BP1 and T47D-BP1 cells had a significant induction of IGFBP-1 transcript compared to EV controls (data not shown).

### Expression of IGF-1R Decreased in MCF7-BP1 and T47-BP1 Cells

**Figure 2:**
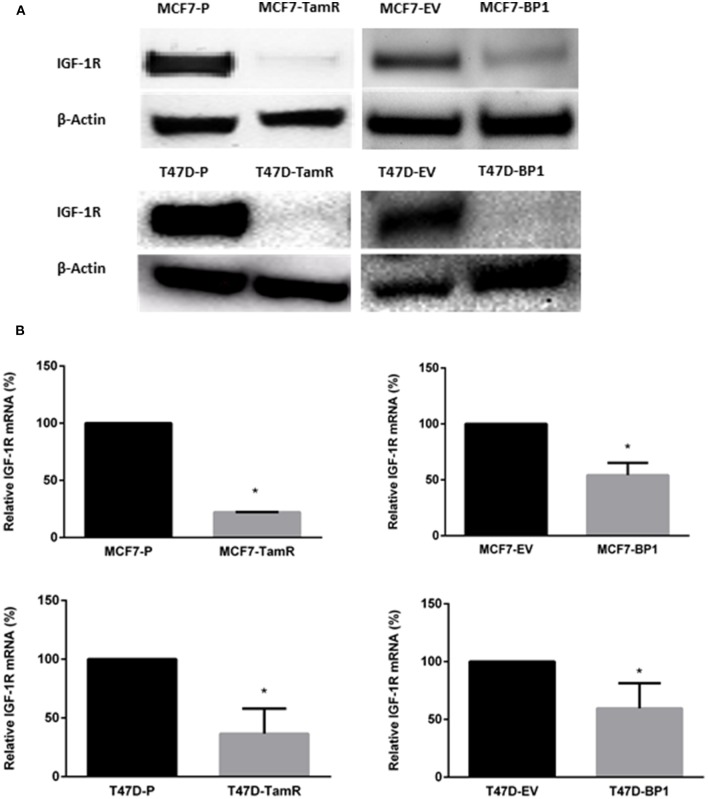
Expression of IGF-1R decreased in MCF7-BP1 and T47-BP1 cells. **(A)** Immunoblot analysis of IGF-1R protein expressions in MCF-7 and T-47D cells. **(B)** qRT-PCR analysis of IGF-1R mRNA levels in MCF-7 and T-47D cells. Results are the average of 3 independent experiments, and error bars are the standard error of the mean. *p < 0.05.

It has been reported that the acquired tamoxifen resistance in MCF-7 and T-47D cells is associated with the decreased IGF-1R transcription and expression (17, 21). In agreement with these reports, the TamR cells generated (Figure 1) expressed significantly less IGF-IR compared to the parental cells. Since IGF-1R expression is associated with tamoxifen resistance, IGF-1R expression was used as an indicator of tamoxifen resistance MCF7-BP1 and T47D-BP1 cells. In both cell lines, IGF-1R expression was decreased compared to MCF7-EV and T47D-EV cells (Figure 2A). Additionally, low levels of IGF-1R transcript were observed in the each TamR cells line when compared to parental cells consistent with previous reports (17). Similar to the observations of decreased IGF-1R expression in TamR breast cancer cells, IGF-1R expression was decreased in MCF7-BP1 and T47D-BP1 compared to MCF7-EV and T47D-EV (Figure 2B). These results suggested that sustained exposure to IGFBP-1 in breast cancer cells contributes to the development of tamoxifen resistance by altering the IGF-1 signaling pathway.

### Sustained IGFBP-1 Exposure Increases EGFR Signaling

**Figure 3:**
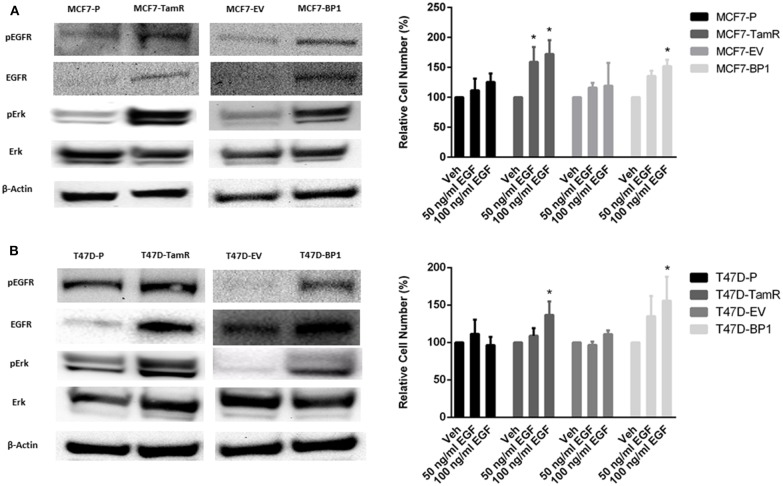
Sustained IGFBP-1 exposure increases EGFR signaling. **(A)** Immunoblot analysis of phospho-EGFR (Y1068), EGFR, phospho-Erk, and Erk in MCF-7 cells (top); measurement of relative cell number (%) in MCF-7 cells after 5 days of EGF treatment (bottom). **(B)** Immunoblot analysis of phospho-EGFR (Y1068), EGFR, phospho-Erk, and Erk in T-47D cells (top); measurement of relative cell number (%) in T-47D cells after 5 days of EGF treatment (bottom). Results are the average of 3 independent experiments, and error bars are the standard error of the mean. *p < 0.05.

The upregulation of epidermal growth factor receptor (EGFR) is commonly observed in tamoxifen resistance MCF-7 cells (22–25). In addition, the increase of EGFR phosphorylation at tyrosine 1068 has been reported (26). Also, it has been shown that the phosphorylation of Erk is elevated in MCF7-TamR cells (21, 23, 24). In the TamR breast cancer cells developed for this study, alterations in signaling pathways consistent with previous reports were observed (Figure 3A). Additionally, MCF7-BP1 cells expressed higher level of phospho-EGFR, EGFR, and phospho-Erk compared to MCF7-EV (Figure 3A). Given that the expression of EGFR was upregulated in MCF7-TamR and MCF7-BP1, stimulation of cells by EGFR was determined. While the viability of MCF7-P and MCF7-EV was not significantly increased upon EGF treatment, the viability of both MCF7-TamR and MCF7-BP1 was increased by EGF in a dosage-dependent manner (Figure 3B). Additionally, an upregulation of EGFR expression in T47D-TamR compared to T47D-P was observed and phospho-EGFR as well as phospho-Erk observed (Figure 3C). Similar to the T47D-TamR, the levels of EGFR, phospho-EGFR, and phospho-Erk were increased in T47D-BP1 compared to T47D-EV (Figure 3C). Similar to the observations with the MCF-7 and derived cells lines, EGF did not increase the viability of both T47D-P and T47D-EV while EGF treatment increased the viability of both T47D-TamR and T47D-BP1 (Figure 3D). Taken together, these data indicated that the sustained exposure to IGFBP-1 results in increased EGFR signaling in breast cancer cells and this transition to EGF sensitivity is similar to the transition that occurs during development of tamoxifen resistance in breast cancer cells.

### Sustained IGFBP-1 Exposure Results in the Development of Tamoxifen Resistance in Breast Cancer Cells

**Figure 4:**
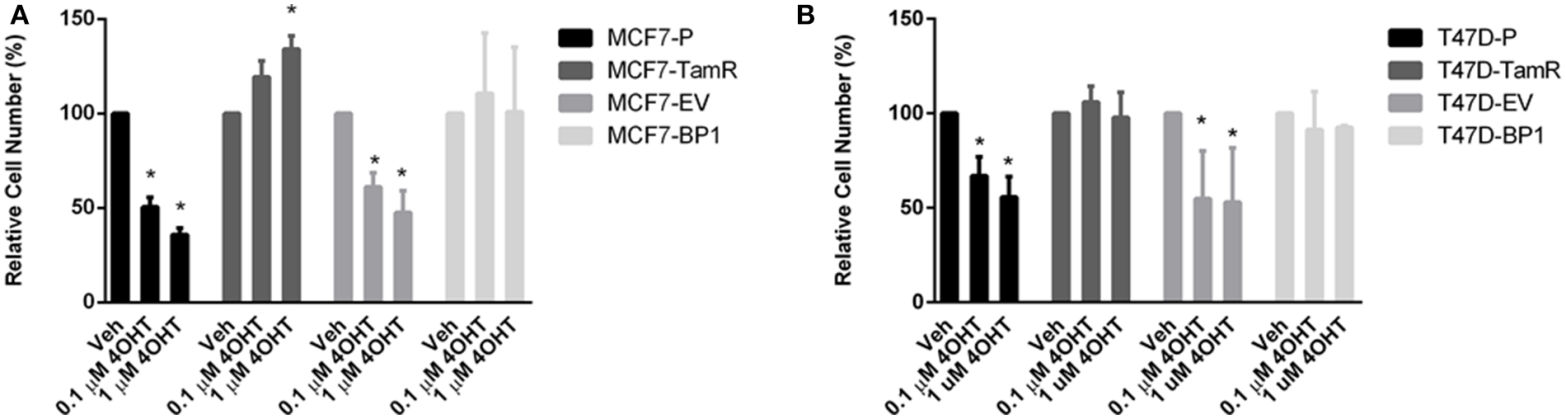
Sustained IGFBP-1 exposure results in the development of tamoxifen resistance in breast cancer cells. **(A)** Measurement of relative cell number (%) in four different MCF-7 cell lines under 4-hydroxytamoxifen (4OHT) treatment for 5 days. **(B)** Measurement of relative cell number (%) in four different T-47D cell lines under 4-hydroxytamoxifen (4OHT) treatment for 5 days. Results are the average of 3 independent experiments, and error bars are the standard error of the mean. *p < 0.05.

EGFR pathway is the predominant pathway related to tamoxifen resistance (27). In particular, it has been suggested that elevated expression of EGFR may serve as an indication of anti-estrogen resistance in ER α positive breast cancer cells (28–30). Given that both of the MCF7-BP1 and T47D-BP1 had higher levels of EGFR, phospho-EGFR, and phospho-Erk, it was hypothesized that the long-term exposure to IGFBP-1 was sufficient for the development of tamoxifen resistance in breast cancer cells. MCF7-BP1 and T47D-BP1 cells were treated with 4-OHT, and cell numbers were measured after 5 days of treatment. While the viability of MCF7-P and MCF7-EV was reduced by the 4-OHT treatment in a dosage-dependent manner, the viability of MCF7-TamR was increased by the 4-OHT treatment, and the viability of MCF7-BP1 was not decreased by 4-OHT (Figure 4). These observations are consistent with previous reports reference previously in this contribution. To determine if the BP-1 variants of the MCF-7 and T-47D cells had a similar resistance to 4-OHT treatment, these cells were treated with 4-OHT and viability was determined by cell counts. Treatment with 4-OHT significantly decreased viability of T47D-P and T47D-EV cells, while cell viability was not significantly altered upon 4-OHT treatment T47D-TamR or T47D-BP1 cells. These data suggest that sustained exposure to IGFBP-1 is sufficient for the development of tamoxifen resistance in breast cancer cells.

### MAPK Inhibition Reverses Tamoxifen Resistance in Breast Cancer Cells

**Figure 5:**
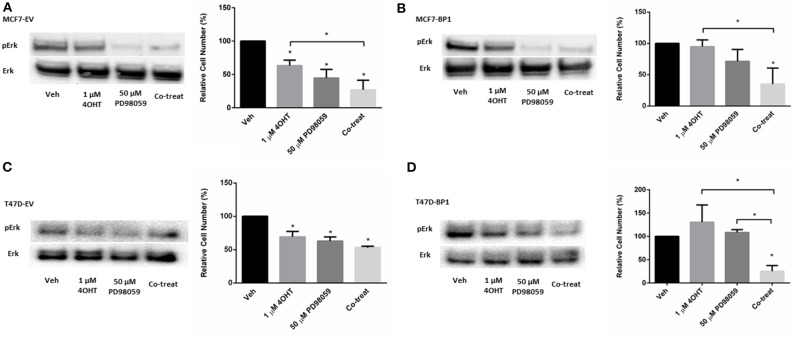
MAPK inhibition reverses tamoxifen resistance in breast cancer cells. **(A)** Left: immunoblot analysis of phospho-Erk (pErk) of treated MCF7-EV; right: measurement of relative cell number (%) of MCF7-EV under different treatments for 5 days. **(B)** Left: immunoblot analysis of phospho-Erk (pErk) of treated MCF7-BP1; right: measurement of relative cell number (%) of MCF7-BP1 under different treatments for 5 days. **(C)** Left: immunoblot analysis of phospho-Erk (pErk) of treated T47D-EV; right: measurement of relative cell number (%) of T47D-EV under different treatments for 5 days. **(D)** Left: immunoblot analysis of phospho-Erk (pErk) of treated T47D-BP1; right: measurement of relative cell number (%) of T47D-BP1 under different treatments for 5 days. Results are the average of 3 independent experiments, and error bars are the standard error of the mean. *p < 0.05.

Activation of Erk plays an important role in the development of tamoxifen resistance in ER α positive breast cancer cells (31–33). Previously, it was reported that MAPK inhibitor PD98059 inhibits proliferation in MCF-7 cells (34), while it had no significant effect in tamoxifen resistant MCF-7 cells (35). However, it was shown that the combination of 4-OHT and PD98059 decreased cell viability tamoxifen resistant MCF-7 cells. To determine if the IGFBP-1 expressing MCF-7 and T-47D cells were sensitive to PD98059 treatment or co-treatment with 4-OHT with PD98059, cell were treated, and viability was determined by cell count. In Figure 5A, the level of phospho-Erk was reduced by PD98059 and the co-treatment of tamoxifen and PD98059 in MCF7-EV. Consistently, the cell numbers of MCF7-EV were significantly decreased by the treatment of 4-OHT and PD98059, and the co-treatment of 4-OHT with PD98050 appeared to have an additive effect on killing MCF7-EV cells (Figure 5A). Both treatment with PD98059 or the co-treatment of 4-OHT with PD98059 effectively reduced the level of phospho-Erk in MCF7-BP1. Interestingly, while neither 4-OHT or PD98050 alone was able to reduce the cell numbers of MCF7-BP1 significantly, the co-treatment of both drugs decreased the viability of MCF7-BP1 cells (Figure 5B). Similar to MCF7-EV, single treatment of 4-OHT or PD98059 significantly reduced the cell numbers of T47D-EV, and the co-treatment of both drugs had an decreased T47D-EV cells (Figure 5C). In T47D-BP1 cells, viability was not reduced by either 4-OHT or PD98059, however co-treatment significantly reduced the viability of T47D-BP1cells (Figure 5D). Taken together, our data revealed that the activation of Erk in MCF7-BP1 and T47D-BP1 cells played a protective role against the 4-OHT treatment, and the inhibition of Erk activation by PD98059 re-sensitized the cells to 4-OHT suggesting that Erk activation in MCF7-BP1 and T47D-BP1 cells was a key element for tamoxifen resistance.

### Knockdown of IGFBP-1 in MCF7-BP1 and T47D-BP1 Reduced the Level of Phospho-Erk and Sensitized the Cells to 4-OHT

**Figure 6:**
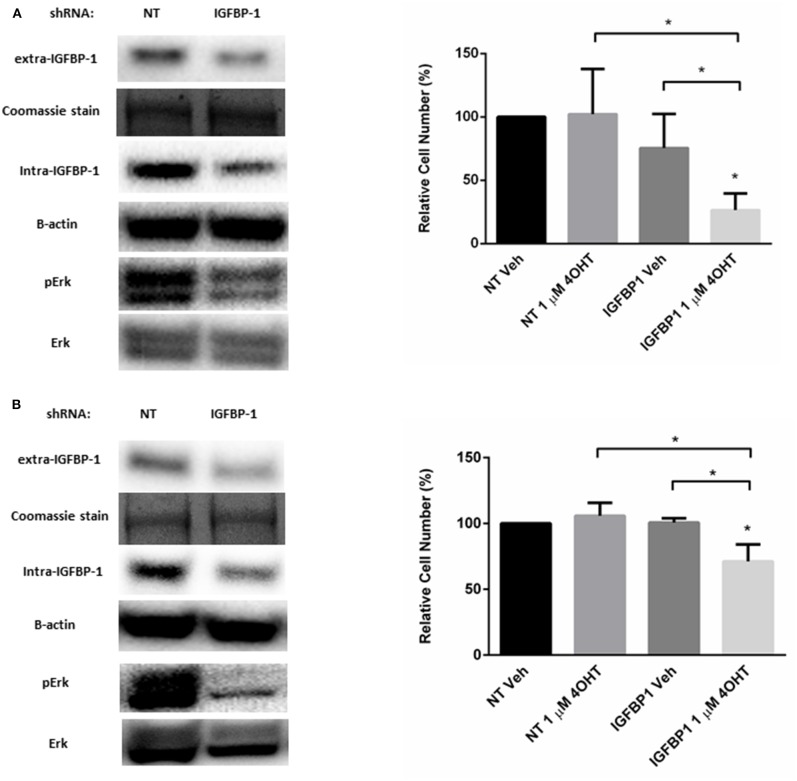
Knockdown of IGFBP-1 in MCF7-BP1 and T47D-BP1 reduced the level of phospho-Erk and sensitized the cells to 4-OHT. **(A)** Immunoblot analysis of IGFBP-1, phospho-Erk, and Erk after transfected with non-targeting (NT) shRNA or IGFBP-1 shRNA in MCF7-BP1 (left); measurement of relative cell number (%) in MCF7-BP1 after transfected with NT shRNA or IGFBP-1 shRNA and treated with either vehicle or 1 μM 4-OHT (right). **(B)** Immunoblot analysis of IGFBP-1, phospho-Erk, and Erk after transfected with non-targeting (NT) shRNA or shRNA for IGFBP-1 in T47D-BP1 (left); measurement of relative cell number (%) in T47D-BP1 after transfected with NT shRNA or IGFBP-1 shRNA and treated with either vehicle or 1 μM 4-OHT (right). Results are the average of 3 independent experiments, and error bars are the standard error of the mean. *p < 0.05.

Besides functioning to regulate IGF-1 action, IGFBP-1 is reported to be a stimulator of Erk in several cell types (36–38). A similar role for IGFBP-1 in breast cancer cells has not been reported. To determine if IGFBP-1 exposure results in the activation of Erk in breast cancer cells, IGFBP-1 was transiently reduced in MCF7-BP1 and T47D-BP1 cells and Erk phosphorylation was measured. The transient knockdown of IGFBP-1 for 96 h effectively reduced both extracellular and intracellular IGFBP-1 accumulation and this knockdown also reduced the accumulation of phospho-Erk in both MCF7-BP1 and T47D-BP1 cells (Figures 6A,B). The previous experiments demonstrated that inhibition of phosphor-Erk accumulation sensitized both MCF7-BP1 and T47D-BP1 cells to 4-OHT treatment (Figure 5B), it was reasoned that knockdown of IGFBP-1 would similarly sensitize MCF7-BP1 and T47D-BP1 cells to 4-OHT treatment. Knockdown of IGFBP-1 in MCF7-BP1 resulted in a result in a significant decrease in viability when treated with 1 μM 4-OHT compared to non-targeting control. Similar to MCF7-BP1 cells, neither the treatment of 4-OHT nor the knockdown of IGFBP-1 decreased the cell numbers of T47D-BP1, whereas the combination of 4-OHT treatment with IGFBP-1 knockdown significantly reduced the cell numbers (Figure 6B). These data suggest that exposure to IGFBP-1 is involved in the development of tamoxifen resistance in breast cancer cells. Furthermore, these data suggest that elevated IGFBP-1 levels stimulate Erk activation and resulting in tamoxifen resistance in breast cancer cells.

### Transient Knockdown of IGFBP-1 Restores Tamoxifen Sensitivity in Breast Cancer Cells

**Figure 7:**
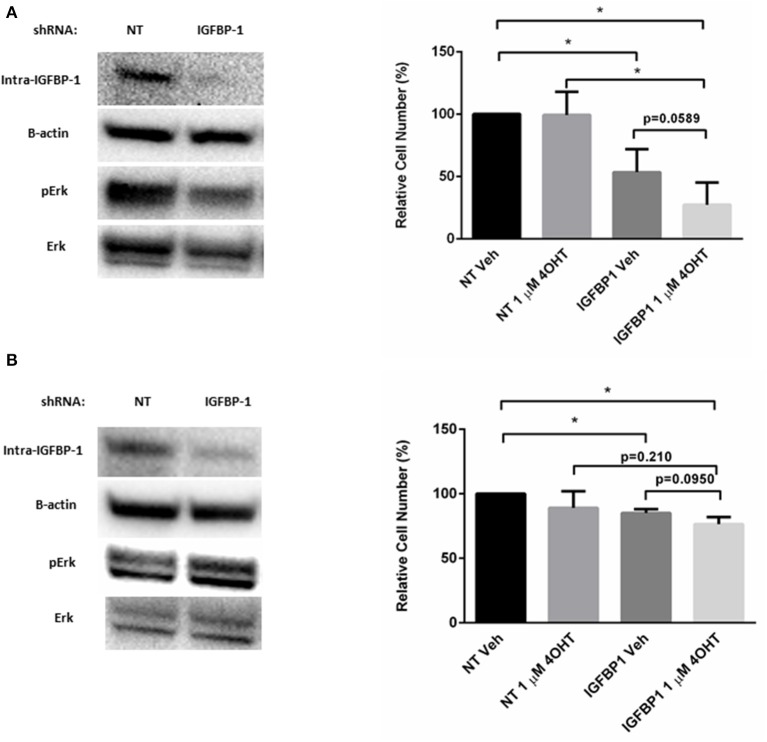
Knockdown of IGFBP-1 restores 4-OHT sensitivity in breast cancer cells. **(A)** Immunoblot analysis of IGFBP-1, phospho-Erk, and Erk after transfected with non-targeting (NT) shRNA or IGFBP-1 shRNA in MCF7-TamR (left); measurement of relative cell number (%) in MCF7-TamR after transfected with NT shRNA or IGFBP-1 shRNA and treated with either vehicle or 1 μM 4-OHT (right). **(B)** Immunoblot analysis of IGFBP-1, phospho-Erk, and Erk after transfected with non-targeting (NT) shRNA or shRNA for IGFBP-1 in T47D-TamR (left); measurement of relative cell number (%) in T47D-TamR after transfected with NT shRNA or IGFBP-1 shRNA and treated with either vehicle or 1 μM 4-OHT (right). Results are the average of 3 independent experiments, and error bars are the standard error of the mean. *p < 0.05.

To determine if elevated IGFBP-1 expression is required for tamoxifen sensitivity in breast cancer cells, transient knockdown of IGFBP-1 expression was performed in both MCF7-TamR and T47D-TamR cells. Transient knockdown of IGFBP-1 for 96 h in MCF7-TamR sufficiently reduced the accumulation of IGFBP-1 and phospho-Erk (Figure 7). Transient knockdown of IGFBP-1 reduced cell viability suggesting that IGFBP-1 is a prosurvival factor in MCF-TamR cells. Furthermore, transient knockdown of IGFBP-1 in MCF7-TamR cells restored tamoxifen sensitivity as indicated by decreased cell numbers upon 4-OHT treatment preceded by IGFBP-1 knockdown. Similar experiments were performed in T47D-TamR, however, IGFBP-1 knockdown was less robust in this cell line. In the T47D-TamR phospho-Erk accumulation was not reduced upon IGFBP-1 knockdown while the knockdown did significantly reduced cell viability of T47D-TamR. These data support the conclusion IGFBP-1 is a pro-survival signal in TamR breast cancer cells. When T47D-TamR were treated with 1 μM 4-OHT after IGFBP-1 knockdown, a reduction of viability was observed, however this reduction was not statistically significant (*p* = 0.0950) like what was observed in the MCF-7 model. Taken together, these results provide evidence that IGFBP-1 contributes to the development of tamoxifen resistance in breast cancer cells and is a pro-survival signal for tamoxifen resistant breast cancer cells.

## Discussion

Tamoxifen resistance remains a clinically relevant complication for women receiving adjuvant breast cancer treatment. Much work has been directed toward understanding tamoxifen resistance and several mechanisms or chemoresistance have been proposed. These range from the loss or alteration of ER α expression to the activation of alternative growth factor pathways observed in tamoxifen resistant cells (39–41). The data described in this contribution provides a link between the G protein-coupled estrogen receptor 1 (GPER1)-mediated IGFBP-1 accumulation associated with tamoxifen treatment in breast cancer cells (15) with the alteration in growth factor signaling previously reported (17). Furthermore, these data demonstrate that sustained IGFBP-1 exposure results in tamoxifen resistance and IGFBP-1 expression is a critical component of chemoresistance in breast cancer cells. Taken together, these data provide support for the conclusion that IGFBP-1 is sufficient to confer tamoxifen resistance in breast cancer cells and is a prosurvival factor for chemoresistant breast cancer cells.

IGFBPs are known to have many functions in cells and these can be intracellular and/or extracellular. Complete elucidation of the role that IGFBP-1 plays in tamoxifen resistance will require continued discovery and analysis of IGFBP-1-mediated cellular pathways. IGFBP-1 is anti-proliferative for MCF-7 cells (14) and T-47D cells (42) was demonstrated to inhibit the mobility of human metastatic breast cancer cell line MDA-231BO (43). Data from these reports supports a tumor suppressive role for IGFBP-1 in breast cancer cells. However, in the tamoxifen resistant breast cancer cell the role for IGFBP-1 has been altered. The results reported herein suggest that IGFBP-1 has a prosurvival role in the tamoxifen resistant breast cancer cell and that sustained IGFBP-1 exposure is sufficient for the development tamoxifen resistance. Thus far, the mechanism by which IGFBP-1 acts to enhance cell viability in breast cancer cells has not been determined. One possible mechanism that underlies the prosurvivla role for IGFBP-1 in breast cancer cells is the known interaction with integrin α5β1. The integrin recognition sequence Arg-Gly-Asp (RGD) of IGFBP-1 interacts with integrin α5β1 resulting in the activation of Erk in multiple cell lines (36–38). Integrin α5β1 has been implicated in solid tumors, and it was shown to promote the adhesion and invasion for breast cancer cells (44).

In addition to the role that IGFBP-1 has in the activation of cellular pathways, the regulation of IGFBP-1 expression and activity will need to be investigated to include analyzing the phosphorylation status of IGFBP-1 in the tamoxifen resistant breast cancer cell context. There are three major sites of phosphorylation in the linker domain of human IGFBP-1, which are Ser 98, Ser 101, and Ser 119 (45). The phosphorylation on these residues contributes to increased binding affinity of IGFBP-1 to IGF-1 (46). Non-phosphorylated IGFBP-1 has lower IGF-1 binding affinity and thus potentiates IGF-1R activation (8) which was also demonstrated to activate Erk (47, 48). One explanation for the data presented here is that IGFBP-1 is expressed but not phosphorylated and therefore potentiates Erk activation in tamoxifen resistant breast cancer cells. This line of investigation will require analysis of the kinase involved in IGFBP-1 phosphorylation such as CK1 and CK2 (49).

Differentiating the extracellular and intracellular roles of IGFBP-1 is necessary to fully understand the contribution of IGFBP-1 in breast cancer cells both during tamoxifen treatment and in the tamoxifen resistant state. For example, IGFBP-3, the most widely studied IGFBP family member, can be internalized and relocated to nucleus via its binding to transferrin and caveolin 1 (50). Once located in the nucleus, IGFBP-3 was shown to interact with histone-DNA complex and act as a transcriptional regulator for certain genes (51). Because IGFBP-1 is expressed both intracellularly and extracellularly and shares high degree of homology of transferrin and caveolin 1 binding regions with IGFBP-3 (50). Until know, the only functions associated with IGFBP-1 in tamoxifen treated breast cancer cells have focused on the extracellular role of this protein. The potential involvement of intracellular IGFBP-1 and the contribution to tamoxifen resistance will need to be studied.

Taken together, these data reveal a novel role for IGFBP-1 in the development of tamoxifen resistance in breast cancer cells. In tamoxifen-sensitive cells, IGFBP-1 accumulation function to decrease cell viability while the long-term exposure to IGFBP-1 results in tamoxifen resistance. This suggests that IGFBP-1 is sufficient for the development of tamoxifen resistance. Furthermore, tamoxifen resistant cells have increased IGFBP-1 accumulation the viability of the cells is decreased when IGFBP-1 is reduced. This suggests that IGFBP-1 is a key prosurvival factor in the tamoxifen resistant cell state. These results may have clinical implications due to the possibility of monitoring IGFBP-1 expression as a marker of tamoxifen resistance.

## Data Availability Statement

The datasets generated for this study are available on request to the corresponding author.

## Author Contributions

YZ contributed to the conception, the acquisition of data, analysis, and interpretation of the work as well as drafting the work and revision of the manuscript. JS contributed to data acquisition and revision of the manuscript. KH contributed to the conception and interpretation of the work, critical revision of the manuscript, final approval, and agrees to be accountable for all aspects of the work.

## Conflict of Interest

The authors declare that the research was conducted in the absence of any commercial or financial relationships that could be construed as a potential conflict of interest.
